# A bibliometric analysis of acute myocardial infarction in women from 2000 to 2022

**DOI:** 10.3389/fcvm.2023.1090220

**Published:** 2023-07-28

**Authors:** Ming Xu, Fupeng Yang, Bin Shen, Jiamei Wang, Wenhao Niu, Hui Chen, Na Li, Wei Chen, Qinqin Wang, Zhiqing HE, Ru Ding

**Affiliations:** ^1^ Department of Cardiology, Shanghai Changzheng Hospital, Naval Medical University, Shanghai, China; ^2^ Shanghai Cardiovascular Institute of Integrative Medicine, Shanghai, China; ^3^ Department of Cardiology, Shanghai Navy Feature Medical Center, Naval Medical University, Shanghai, China

**Keywords:** women, acute myocardial infarction, citespace, VOSviewer, bibliometrics

## Abstract

**Background:**

Plenty of publications had been written in the last several decades on acute myocardial infarction (AMI) in women. However, there are few bibliometric analyses in such field. In order to solve this problem, we attempted to examine the knowledge structure and development of research about AMI in women based on analysis of related publications.

**Method:**

The Web of Science Core Collection was used to extract all publications regarding AMI in women, ranging from January 2000 to August 2022. Bibliometric analysis was performed using VOSviewer, Cite Space, and an online bibliometric analysis platform.

**Results:**

A total of 14,853 publications related to AMI in women were identified from 2000 to 2022. Over the past 20 years, the United States had published the most articles in international research and participated in international cooperation the most frequently. The primary research institutions were Harvard University and University of Toronto. Circulation was the most cited journal and had an incontrovertible academic impact. 67,848 authors were identiﬁed, among which Harlan M Krumholz had the most signiﬁcant number of articles and Thygesen K was co-cited most often. And the most common keywords included risk factors, disease, prognosis, mortality, criteria and algorithm.

**Conclusion:**

The research hotspots and trends of AMI in women were identified and explored using bibliometric and visual methods. Researches about AMI in women are ﬂourishing. Criteria and algorithms might be the focus of research in the near future, which deserved great attentions.

## Introduction

Cardiovascular disease (CVD) has become the leading cause of death among women around the world (1). Despite of striking improvements in decreasing cardiovascular mortality among women over the past 20 years (2), the exact pathological mechanism of coronary artery disease (CAD) still remained elusive, and misdiagnosis and improperly treatment is prevalent among this vulnerable population. There is a growing body of evidence of sex differences in risk factors, coronary artery anatomy and function, symptom presentation, comorbidities, and treatment outcomes, but the involved mechanisms are still unclear (3, 4). The knowledge gaps are partly due to the underrepresentation of female patients in cardiovascular clinical trials and a lack of basic research work, which might be caused by a general concern that the inclusion of females would increase the variability of the trial results, as well as the sample size and cost (5–9). Although the obstructive atherosclerotic disease of the epicardial coronary arteries remained the underlying cause of AMI in both sexes, women are shown to be more likely to present with myocardial infarction without obstructive coronary artery disease (MINOCA) (10, 11). In addition to coronary microvascular dysfunction, other coronary causes of MINOCA include coronary vasodilatory disorders, non-atherosclerotic spontaneous coronary artery entrapment (12), and coronary thrombosis/embolism. Patients with CAD (even without obstruction) had a worse prognosis, in which the atherosclerotic burden might play a key role (13, 14). The severity of epicardial coronary artery obstructive disease appeared lower in women than those in men during selective angiography despite of higher burden of risk factors and angina symptoms (15). Multiple studies had shown that compared with men with acute coronary syndrome (ACS), female patients were less likely to be prescribed with guideline-directed medications and receive intravascular intervention (16–19). Furthermore, women are more likely to have longer prehospital delay in presentation after symptom onset, as well as underdiagnosis of AMI and lower priority for emergency ambulance services (17, 18, 20–24). So female patients are shown to present with more other symptoms and suffer from a higher in-hospital mortality rate than male patients (25, 26). The difference in mortality was particularly pronounced among patients under 55 years of age (27). So decreasing the morbidity and mortality of CAD and closing the knowledge gap on clinical presentation and treatment of AMI in women have become the public health priorities.

The purpose of this study was to perform a bibliometric analysis of publications on AMI in women over the past 20 + years (from 2000 to 2022) to identify key contributors and the current state of researches and to reveal emerging areas of such field, which might be helpful to recognize the sex gap and achieve gender equity in AMI management and outcomes in the future.

## Methods

### Data sources and search strategy

**Figure 1:**
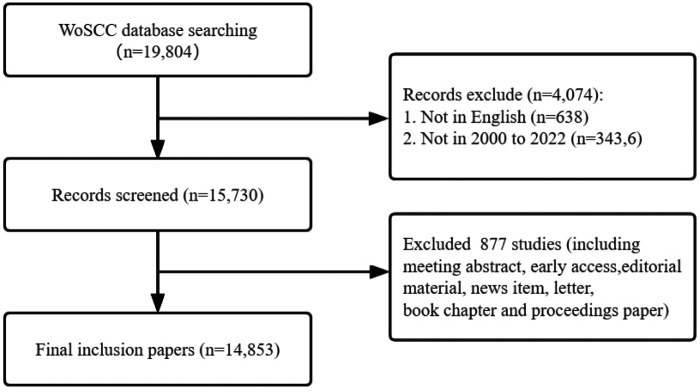
Flowchart of data filtration processing and excluding publications.

All data were retrieved from the Web of Science Core Collection (WoSCC). To ensure comprehensive and accurate retrieval data, the indexes included SSCI and SCI-EXPANDED. The retrieval formula was shown as the following (TS = (acute myocardial infarction) AND (((([TS = (sex)] OR TS = (Female)) OR TS = (woman)) OR TS = (women)) OR TS = (gender))), and the period was set from 2000 to 01-01 to 2022-08-01. The enrolled article types included Articles and Review Articles, and the language was limited to English. The search results were exported with “Plain Text file” and the record content chose “Full Record and Cited References”, and stored in download_*.txt format (Figure 1).

### Data analysis and visualization

All valid data were collected from Web of Science Core Collection and imported to Microsoft Excel 2016, VOSviewer, and CiteSpace for further visual analysis.

VOSviewer (version 1.6.18) is a bibliometric analysis software that can extract the key information from numerous publications (28), which is often used to build collaboration, co-citation, and co-occurrence networks (29, 30). In our study, the software mainly completed the following analysis: country and institution analysis, journal and co-cited journal analysis, and keyword co-occurrence analysis. In the map produced by VOSviewer, a node represents an item such as country, institution, and journal. Node size and color indicate the number and classiﬁcation of these items, respectively. Line thickness between nodes reﬂects the degree of collaboration or co-citation of the items (31, 32).

CiteSpace (version 5.8.R3) is another software developed by Professors Chen C for bibliometric analysis and visualization (30, 33), which was applied to map the dual-map overlay of journals, analyze author, co-cited authors and references with Citation Bursts.

The R package “bibliometrix” (version 4.0.0) (https://www.bibliometrix.org) was applied for a thematic evolution analysis of AMI in women (34). The quartile and impact factors of the analyzed journals were obtained from Journal Citation Reports 2021.

## Results

### Trends and annual publications

**Figure 2:**
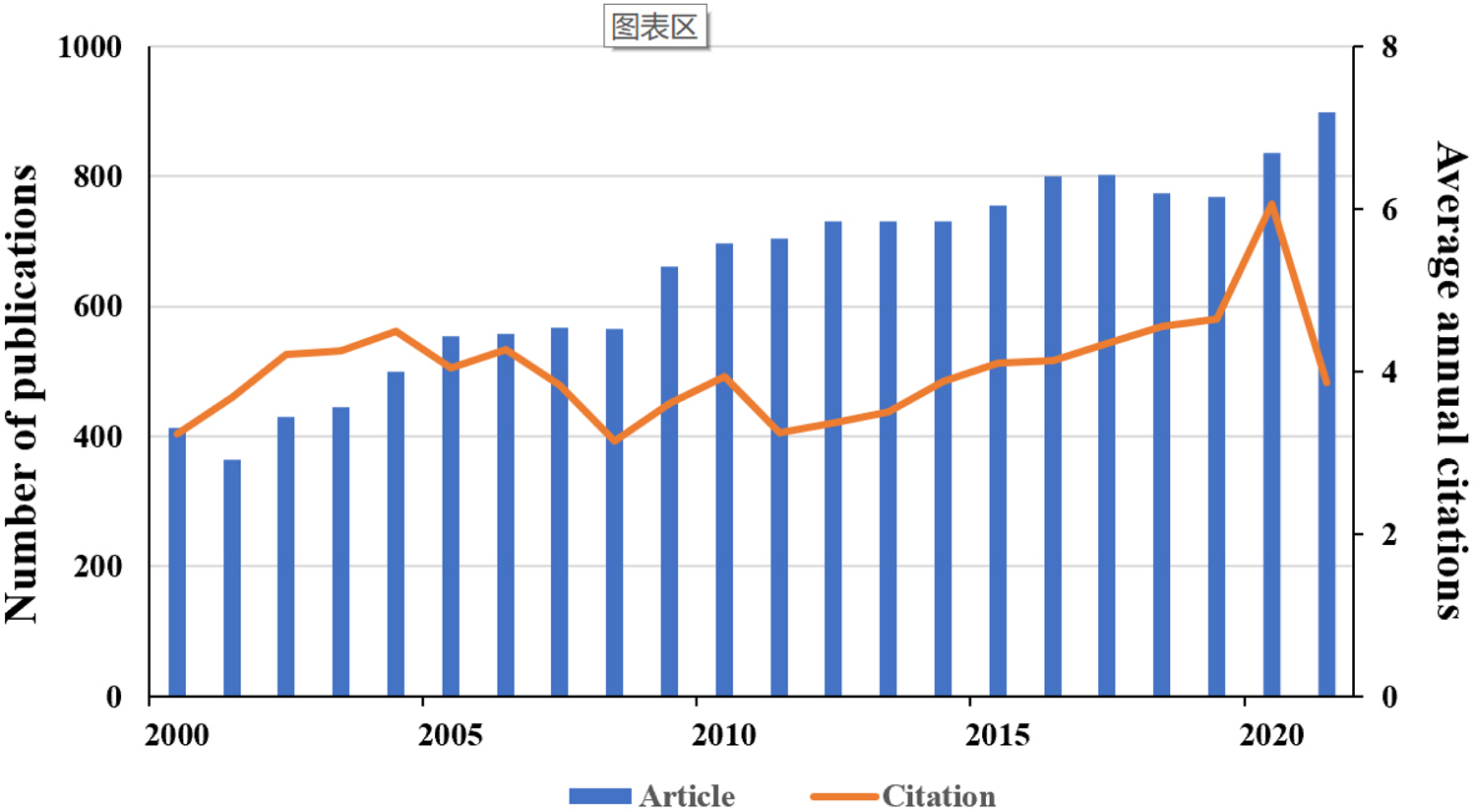
Number of annual publications and average annual citations.

According to the preset search strategy, a total of 14,853 studies on AMI in women in the past two decades, including 13,871 “articles” and 982 “reviews” were identified. The annual output growth is indicative of trends in specific areas of research. Figure 2 shows the annual number of publications and the average number of citations per year in the field of AMI in women. Overall, there is a steady growth trend in this area. The article output was 414 in 2000, while the production peak appeared in 2021, in which 899 articles were published and the annual growth rate reached 3.67%. The average annual citation also tended to stabilize with a phase burst around 2021.

### Contribution of countries and institutions

**Figure 3:**
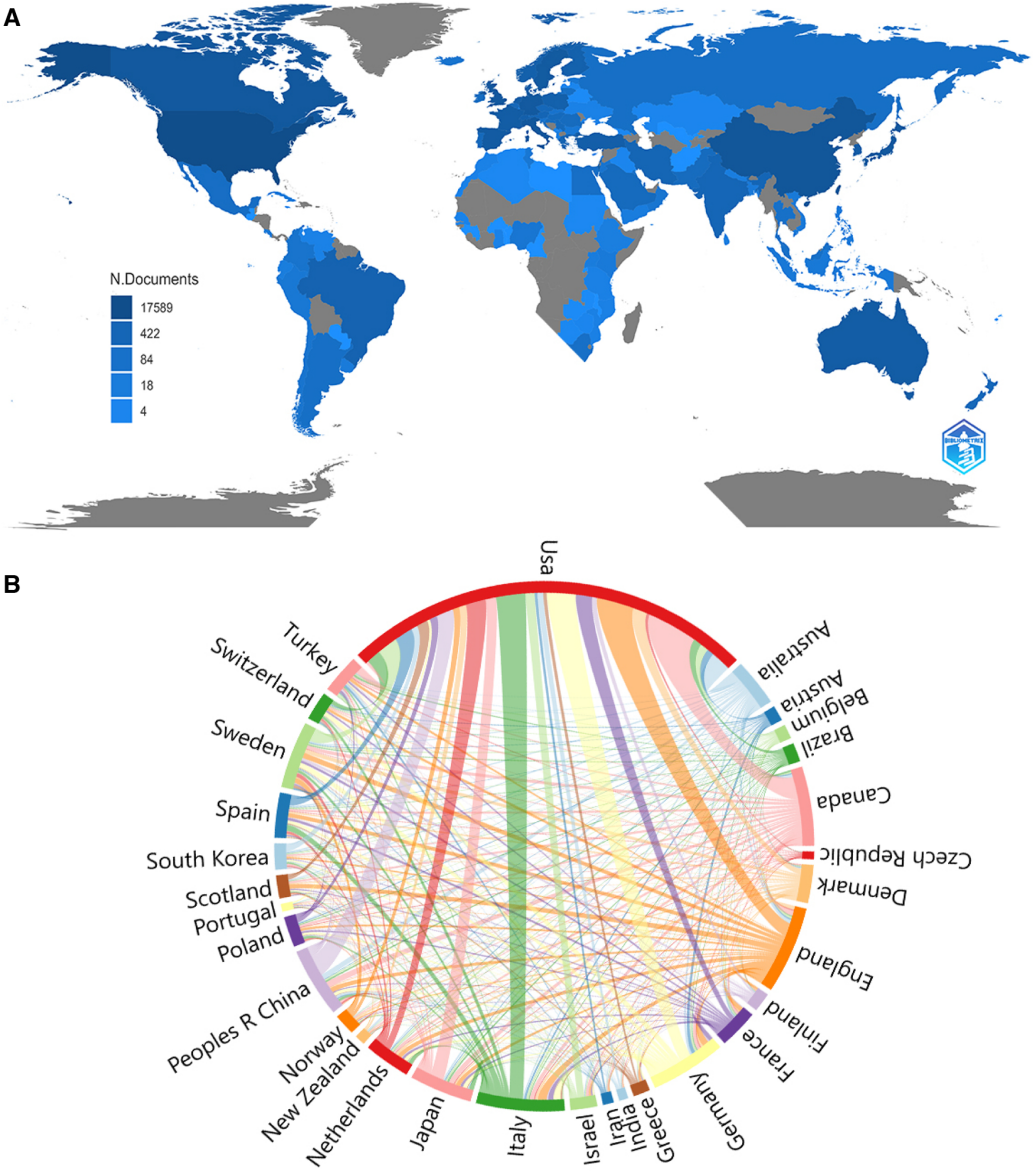
The geographical distribution (**A**) and visualization of countries (**B**) on the research of AMI in women.

According to the WoSCC database, 127 countries or regions contributed to researches about AMI in women from 2000 to 2022. The United States (*n* = 5,142) had the highest number of publications in the field of AMI in women, followed by England (*n* = 1,103), Italy (*n* = 1,100), Canada (*n* = 1,005), Germany (*n* = 949), China (*n* = 882), Sweden (*n* = 850), Japan (*n* = 784), Australia (*n* = 598), and the Netherlands (*n* = 582). The combined number of publications from the United States and England accounted for almost half of the total (42.02%) (Table 1). Subsequently, 30 countries were filtered and visualized based on the number of publications more than or equal to 100, and a collaborative network was constructed based on the number and relationship of publications in each country (Figure 3). Notably, plenty of active cooperations were shown among different countries. For example, experts from The United States had close cooperations with those from China, Canada, and Japan; and those from Germany was shown to keep active cooperations with those from Australia, France, Brazil, and Greece. Need to acknowledge, there are still scientifically relevant publications from countries who are not listed in the most frequently published countries. If there are cross-country publications, the country of publication is usually determined by the corresponding author.

**Table 1:** Top 10 countries and institutions on the research of AMI in women.

Rank	Countries			Institutions		
	Countries	Article counts	Percentage	Institutions	Article counts	Percentage
1	USA	5142	34.60%	Harvard University	401	2.70%
2	England	1103	7.42%	University of Toronto	302	2.03%
3	Italy	1100	7.41%	Mayo Clinic	294	1.98%
4	Canada	1005	6.77%	Duke University	289	1.95%
5	Germany	949	6.37%	Karolinska Institutet	284	1.91%
6	China	882	5.94%	Brigham & Womens Hospital	261	1.76%
7	Sweden	850	5.72%	Yale University	206	1.39%
8	Japan	784	5.25%	University of California	188	1.27%
9	Australia	598	4.03%	Harvard Medical School	186	1.25%
10	Netherlands	582	3.92%	Emory University	182	1.23%

**Figure 4:**
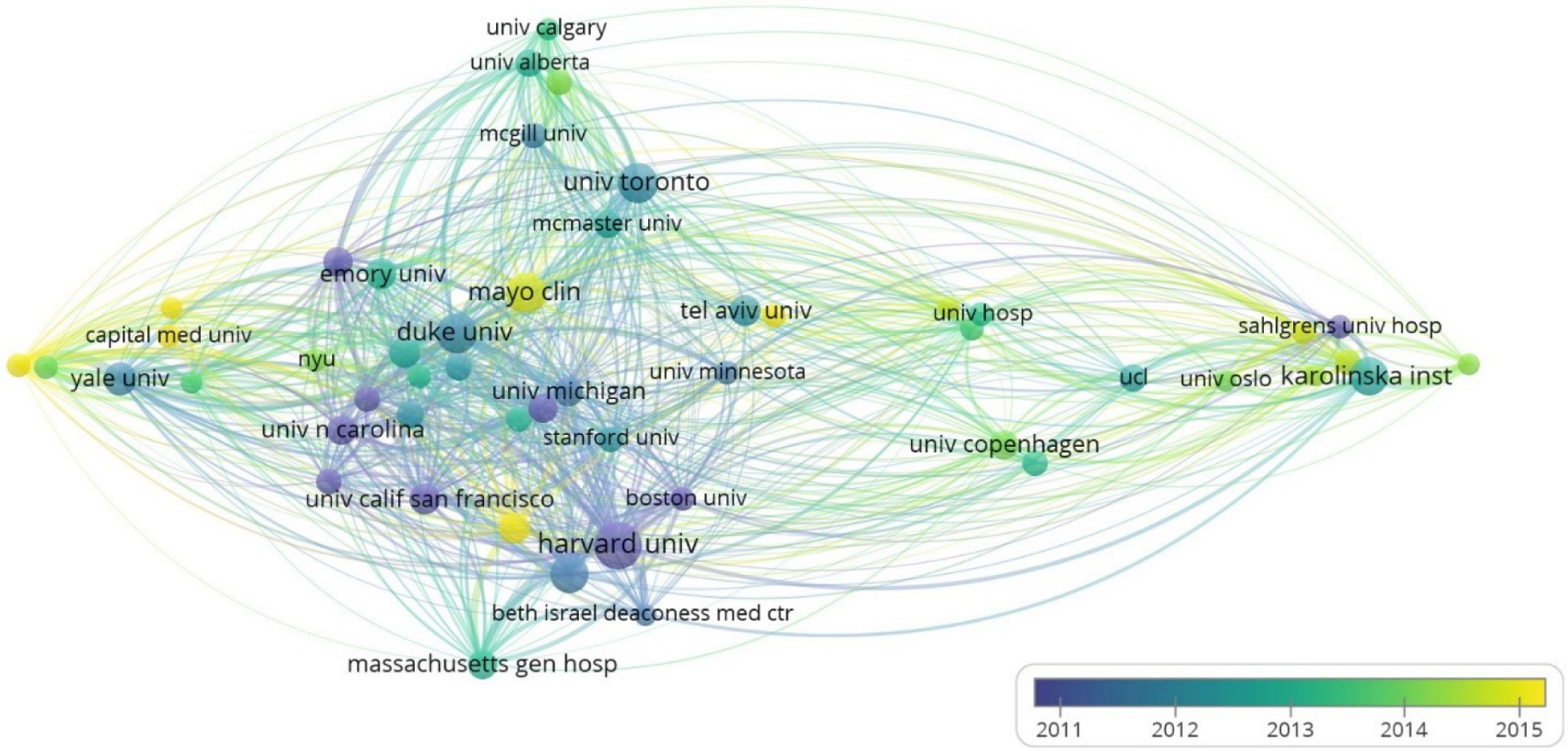
Visualization of institutions involved in the research of AMI in women.

The top 10 institutions were from the USA (8/10), Canada (1/10), and Sweden (1/10). Harvard University (401, 2.70%), University of Toronto (302, 2.03%), Mayo Clinic (294, 1.98%), Duke University (289, 1.95%), and Karolinska Institutet (284, 1.91%) were top 5 productive institutions (Table 1). Subsequently, 51 institutions were selected based on the minimum number of publications equal to 94 for visualization and a collaborative network was constructed (Figure 4). Each circle in the figure represents a country, and the size of the circle indicates the publication outputs by the country. The lines between the circles denote cooperation between countries, and the wider the lines indicate the closer the cooperation. As shown in Figure 4, the cooperations between Duke University, Mayo Clinic, and University of Washington were very close, and there were active cooperations among Harvard University, Harvard Medical School, Brigham and Women's Hospital, and Boston University.

### Journals and co-cited academic journals

**Figure 5:**
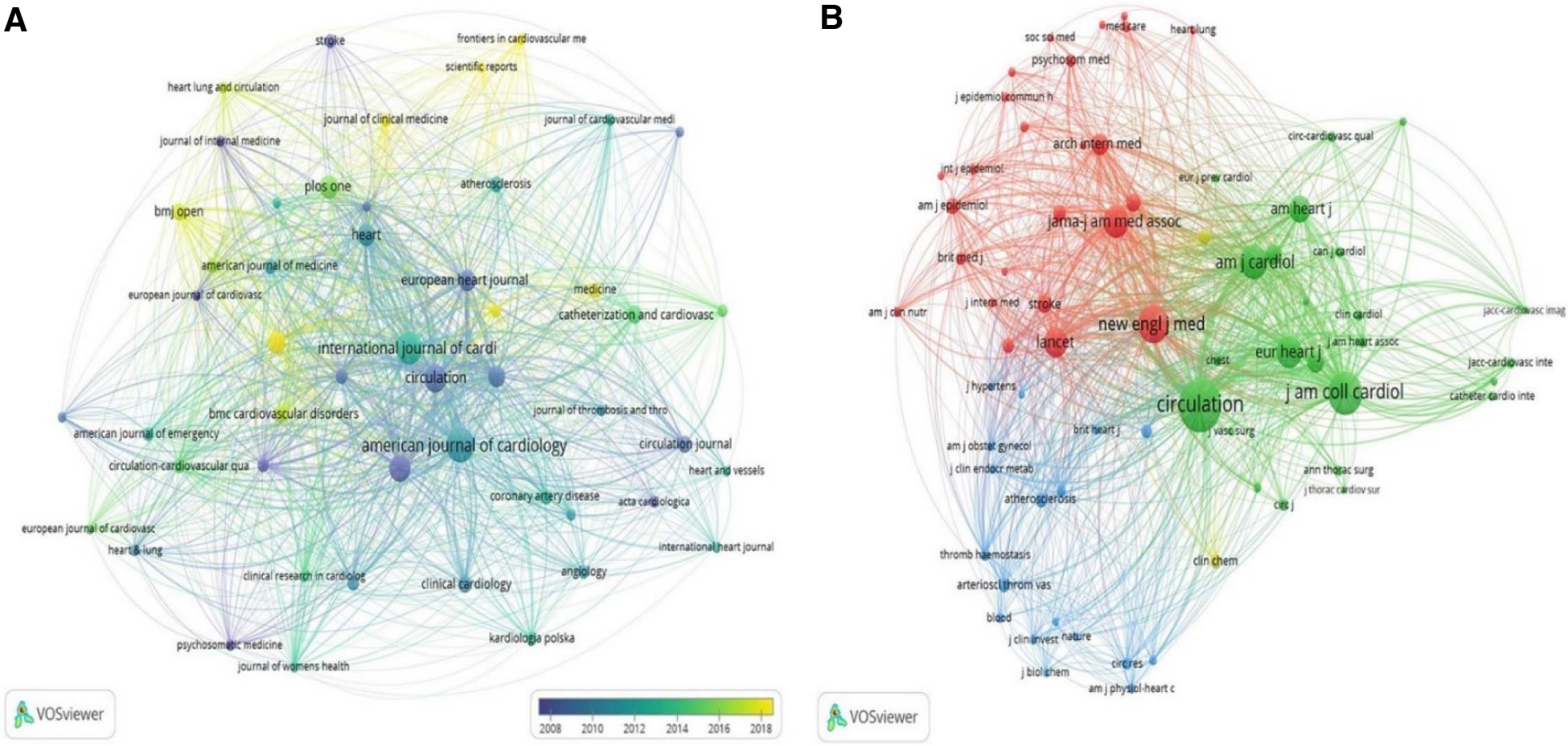
Visualization of journals (**A**) and co-cited journals (**B**) on the research of AMI in women.

A visual analysis of published journals was performed using the VOSviewer software. 14,853 articles involved with AMI in women were shown to be published in 1,867 academic journals, among which *American Journal of Cardiology* (*n* = 522, 3.51%) had the highest number of outputs, followed by *International Journal of Cardiology* (*n* = 462, 3.11%), *Circulation* (*n* = 315, 2.12%) and *American Heart Journal* (*n* = 307, 2.07%). Among the top 10 journals, the journal with the highest impact factor was *Circulation* (IF = 39.92), followed by *European Heart Journal* (IF = 35.85). Furthermore, 30% of journals were shown to belong to Q1 (Table 2). Subsequently, 50 journals were screened based on the minimum number of relevant publications equal to 50 to map the journal network (Figure 5A). It was showed that publications in *Circulation* had active citation relationships with those in I*nternational Journal of Cardiology*, *Cardiology, American Journal of Cardiology*, etc.

**Table 2:** Top 10 journals and co-cited journals related to AMI in women.

Rank	Journal	Count (%)	IF (2021)	JCR	Co-cited journal	Citation	IF (2021)	JCR
1	American Journal of Cardiology	522 (3.51%)	3.133	Q3	Circulation	43138	39.918	Q1
2	International Journal of Cardiology	462 (3.11%)	4.039	Q2	Journal of the American College of Cardiology	27753	27.203	Q1
3	Circulation	315 (2.12%)	39.918	Q1	New England Journal of Medicine	22792	176.079	Q1
4	American Heart Journal	307 (2.07%)	5.099	Q2	American Journal of Cardiology	17855	3.133	Q3
5	Plos One	244 (1.64%)	3.752	Q2	European Heart Journal	16340	35.855	Q1
6	Heart	214 (1.44%)	7.398	Q1	Journal of the American Medical Association	15623	157.335	Q1
7	Journal of the American Heart Association	204 (1.37%)	6.106	Q2	Lancet	14100	202.731	Q1
8	Journal of the American College of Cardiology	203 (1.37%)	4.039	Q2	American Heart Journal	11505	5.099	Q2
9	European Heart Journal	201 (1.35%)	35.855	Q1	Archives of Internal Medicine	6997	17.333	Q1
10	Catheterization and Cardiovascular Interventions	144 (0.97%)	2.585	Q3	International Journal of Cardiology	6946	4.039	Q2

Co-citation analysis is designed to measure the degree of relationship between articles. The impact of a journal depends on its co-citation frequency, which reﬂects the inﬂuence of a journal in a speciﬁc research ﬁeld. As shown in Table 2, among the top 10 co-cited journals, 3 journals were cited more than 20,000 times, and *Circulation* (Co-citation = 43,138) was the most cited journal, followed by *Journal of the American College of Cardiology* (Co-citation = 27,753), *New England Journal of Medicine* (Co-citation = 22,792) and *American Journal of Cardiology* (Co-citation = 17,855). In addition to the above all, the impact factor of Lancet was the highest (IF = 202.73), followed by *New England Journal of Medicine* (IF = 176.08). The co-citation network was mapped by filtering out journals with a minimum co-citation of 1,000 (Figure 5B). As shown in Figure 5B, publications in *European Heart Journal* had positive co-citation relationships with those in *Chest*, *Heart*, *American Journal of Cardiology*, etc.

**Figure 6:**
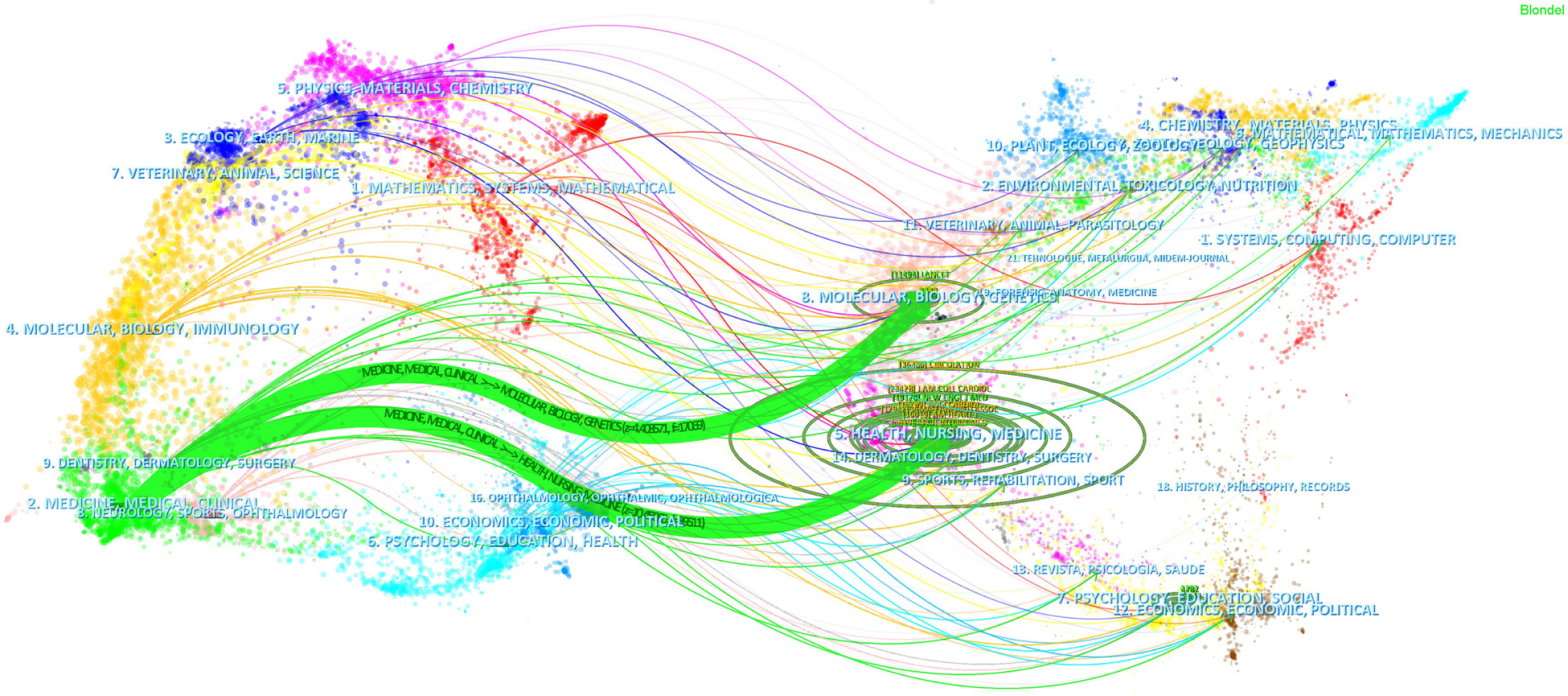
The dual-map overlay of journals on AMI in women.

The dual-map overlay of journals demonstrated relationship distribution among journals, with citing journals on the left and cited journals on the right, and the colored paths between them suggesting the cited relationships. A green path in Figure 6 indicated that the documents published in Health/ Molecular / Biology / Genetics / Nursing journals were often cited by Medical / Clinical ones.

### Authors and co-cited authors

**Figure 7:**
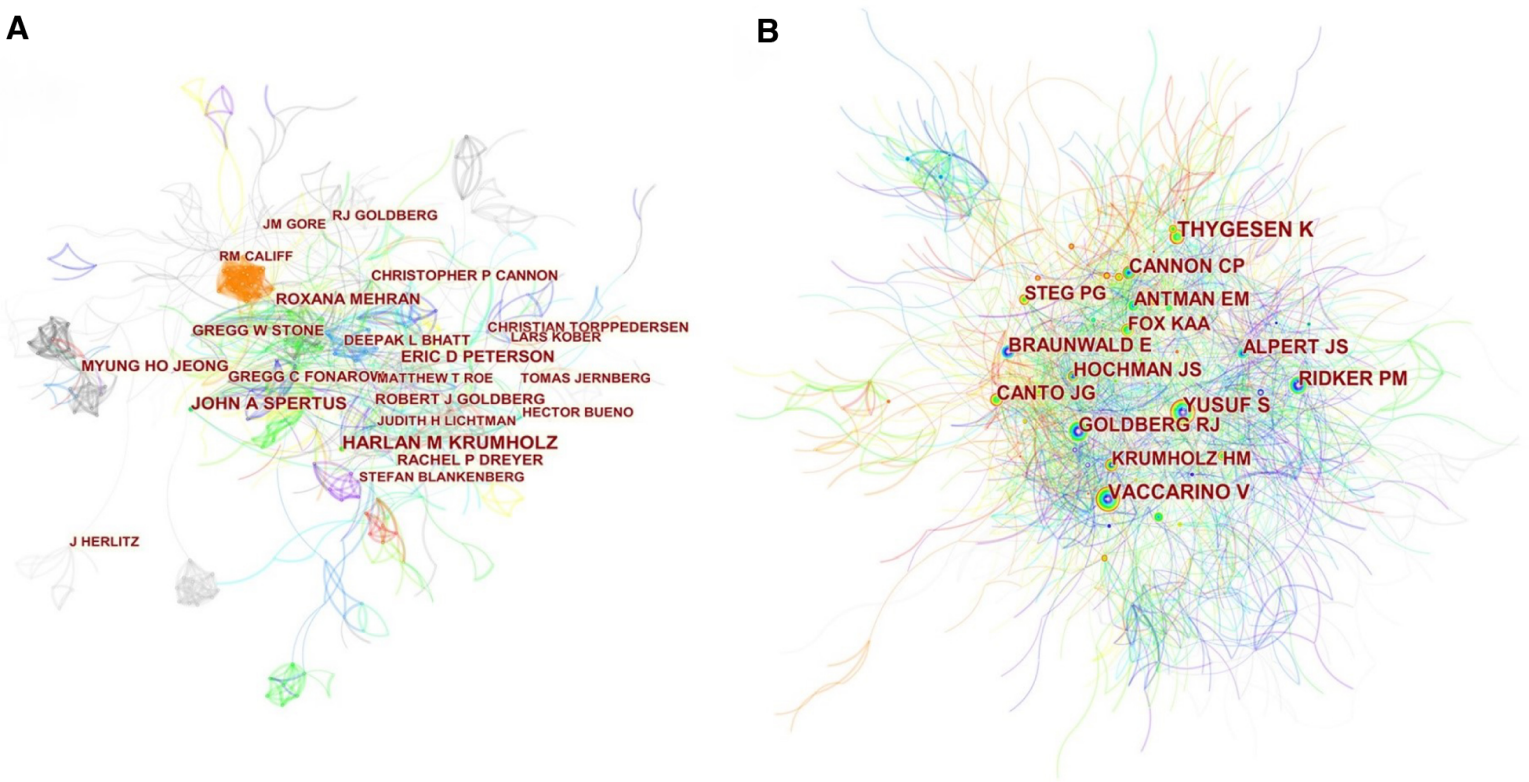
Citespace visualization map of authors (**A**) and co-cited authors (**B**) involved in AMI in women.

A total of 67,848 authors published articles about AMI in women. As shown in Table 3, Harlan M Krumholz from Yale University had the highest number of published papers (*n* = 110, 0.74%), followed by John A Spertus (*n* = 90, 0.61%), Eric D Peterson (*n* = 67, 0.45%), Roxana Mehran (*n* = 57, 0.38%), and Myung Ho Jeong (*n* = 47, 0.32%). Betweenness centrality is an indicator of measuring key nodes in a bibliometric map. Authors in the co-occurrence map showed low centrality (*n* = 0), suggesting that researchers need to engage in further exploration and collaboration on this field. Each circle represents an author, the lines between the circles mean the connections between authors, and the connection network of different colors shows the cooperation cluster between different authors. Figure 7A showed a communication and cooperation network among authors in this research area, such as Harlan M Krumholz, Eric D Peterson, Gregg W Stone, and Robert J Goldberg. Co-cited authors are two or more authors who are cited by another or more papers at the same time, and these two or more authors constitute a co-cited relationship (Figure 7B). Among 146,472 co-cited authors, Thygesen K (Co-citation = 954) was the most frequently cited author, followed by Vaccarino V (Co-citation = 799). 5 co-cited authors had been cited more than 500 times (Table 3).

**Table 3:** Top 10 authors and co-cited authors related to AMI in women.

Rank	Authors	Count (%)	Centrality	Co-cited author	Citation	Centrality
1	Harlan M Krumholz	110 (0.74%)	0.01	Thygesen K	954	0.01
2	John A Spertus	90 (0.61%)	0.04	Vaccarino V	799	0.08
3	Eric D Peterson	67 (0.45%)	0.03	Yusuf S	788	0.02
4	Roxana Mehran	57 (0.38%)	0.05	Ridker PM	618	0.03
5	Myung Ho Jeong	47 (0.32%)	0.04	Goldberg RJ	542	0.04
6	Robert J Goldberg	41 (0.28%)	0.03	Canto JG	470	0.03
7	Rachel *P* Dreyer	40 (0.27%)	0.00	Hochman JS	467	0.03
8	Gregg C Fonarow	39 (0.26%)	0.02	Cannon CP	457	0.03
9	Gregg W Stone	39 (0.26%)	0.06	Antman EM	453	0.04
10	Deepak L Bhatt	38 (0.26%)	0.09	Braunwald E	452	0.02

### Co-cited references and references burst

Co-citation refers to that two or more articles are cited by one or more papers at the same time, and the two articles are considered to be a co-citation relationship. The 10 most frequently cited references were listed. Among the 280,448 co-cited references retrieved, 5 references were cited more than 300 times, and the top three references were all cited more than 350 times (Table 4). The most frequently cited reference topic was *Sex-based differences in early mortality after myocardial infarction*. It was found that the younger the age of the patients, the higher the risk of death among women relative to men. Younger women with myocardial infarction represented a high-risk group deserving of great attentions.

**Table 4:** Top 10 co-cited references related to AMI in women.

Rank	Cited References	DOI	Citations
1	Vaccarino V, 1999, New England Journal of Medicine	10.1056/NEJM199907223410401	440
2	Yusuf S, 2004, Lancet	10.1016/S0140-6736 (04)17018-9	384
3	Roffi M, 2016, European Heart Journal	10.1093/EURHEARTJ/EHV320	355
4	Thygesen K, 2012, Journal of The American College Of Cardiology	10.1016/J.JACC.2012.08.001	315
5	Alpert Js, 2000, Journal of The American College of Cardiology	10.1016/S0735-1097 (00)00804-4	307
6	Steg Pg, 2012, European Heart Journal	10.1093/EURHEARTJ/EHS215	289
7	Ibanez B, 2018, Kardiologia Polska	10.5603/KP.2018.0041	282
8	Wittstein Is, 2005, New England Journal of Medicine	10.1056/NEJMOA043046	271
9	Hochman Js, 1999, New England Journal of Medicine	10.1056/NEJM199907223410402	263
10	Charlson Me, 1987, Journal of Chronic Diseases	10.1016/0021-9681 (87)90171-8	255

**Figure 8:**
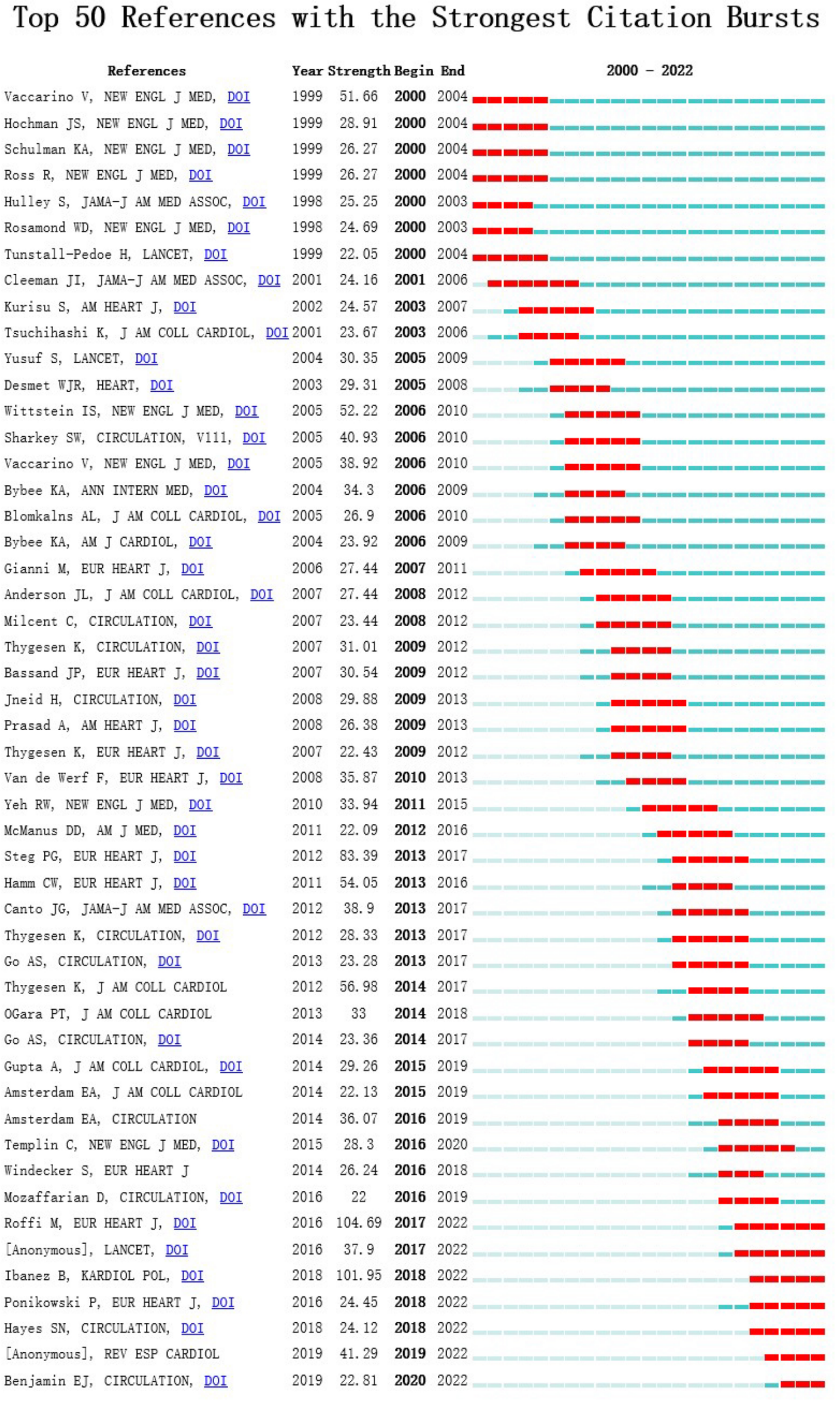
Citespace visualization map of top 50 references with the strongest citation bursts involved with researches about AMI in women.

Reference with citation bursts refers to those references that are frequently cited by scholars in a certain ﬁeld over some time. Figure 8 showed the top 50 references with the most robust citation bursts. The ﬁrst reference with citation burst was published in 2000, and the reference with the strongest citation burst (strength = 104.69) was titled “2015 ESC Guidelines for the management of acute coronary syndromes in patients presenting without persistent ST-segment elevation”, authored by Marco Roffi et al. with citation bursts from 2016 to 2017.

### Hotspots and frontiers

**Figure 9:**
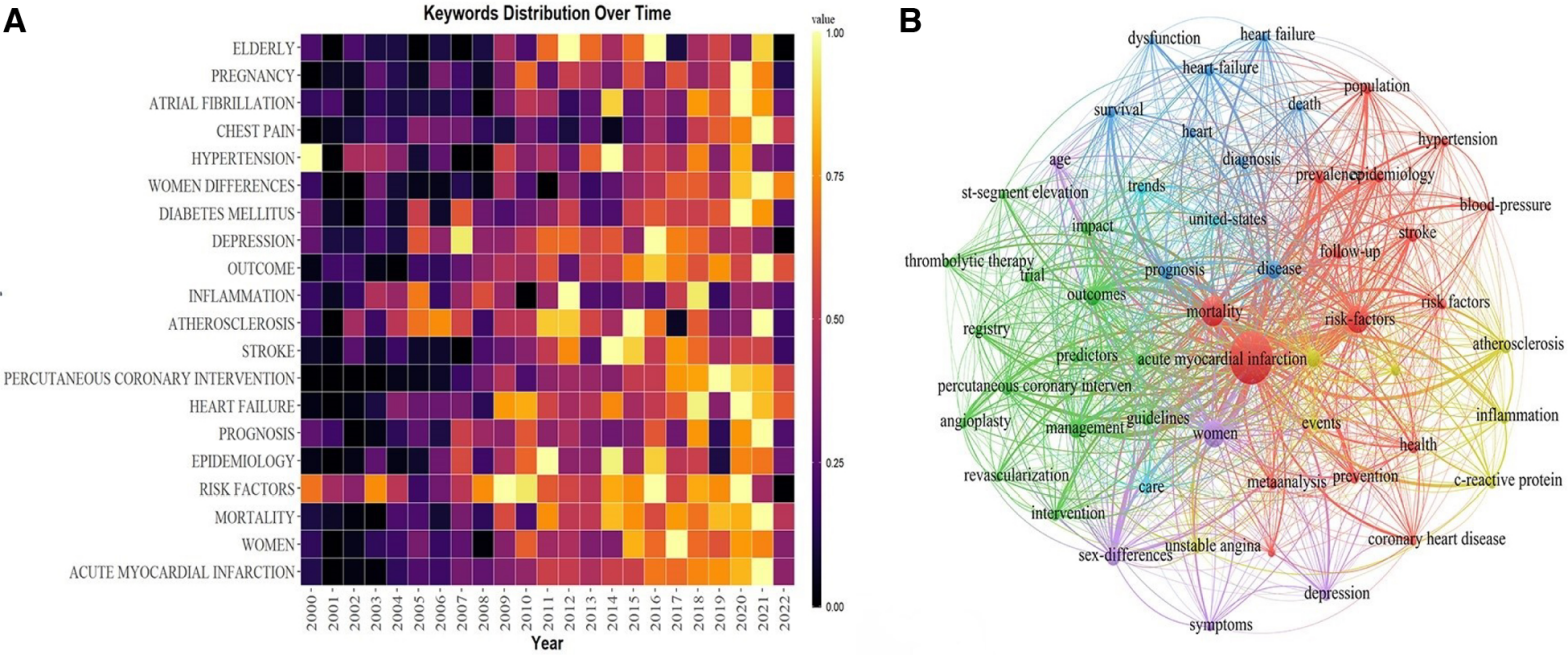
(**A**) the top 20 author keywords distribution over time. (**B**) The visualization of keywords network.

Keywords summarize research topics. Through the analysis of keywords, we can understand the research hotspots in speciﬁc ﬁelds and explore the hotspots and research directions. Table 5 showed the top 20 high-frequency keywords in research of AMI in women. Among these keywords, mortality and risk factors appeared more than 3,000 times, which represented the main research direction of AMI in women. Figure 9A showed the top 20 high-frequency author keyword distribution over time of AMI in women, which visually reﬂected the dynamic changes of hotspots and developmental path of AMI in women’s research.

**Table 5:** Top 20 keywords on the research of AMI in women.

Rank	Keywords	Count	Rank	Keywords	Count
1	Acute myocardial infarction	11458	11	Atherosclerosis	772
2	Mortality	3592	12	Percutaneous coronary intervention	762
3	Risk-factors	3163	13	Stroke	747
4	Women	2889	14	Prevalence	739
5	Outcomes	1463	15	Impact	661
6	Management	1412	16	Survival	658
7	Disease	1383	17	Epidemiology	649
8	Association	1216	18	Heart-failure	620
9	Sex-differences	1198	19	Population	584
10	Prognosis	837	20	Therapy	576

54 keywords were filtered with a number of occurrences more than or equal to 306 and subjected cluster analysis through VOSviewer (Figure 9B). The larger the dot in the figure represents the more times the keyword appears, the more representative of the field hotspots; the node connection represents the strength of association, and the thicker the line indicates that the two appear together in the same literature more times; the color of the dot represents different clusters. Six clusters represented six different research directions (Figure 9B). The keywords in the red cluster were shown as acute myocardial infarction, blood pressure, epidemiology, mortality, population, etc. Impact, management, outcome, percutaneous coronary intervention, therapy, etc were included in the green cluster. While death, diagnosis, disease, dysfunction, prognosis, heart failure, etc were found in the blue cluster. And association, atherosclerosis, c-reactive protein, events, inflammation, and unstable angina were shown in the yellow one. While the purple one contained age, sex differences, depression, and symptoms, and the pale blue cluster mainly included care and trends.

**Figure 10:**
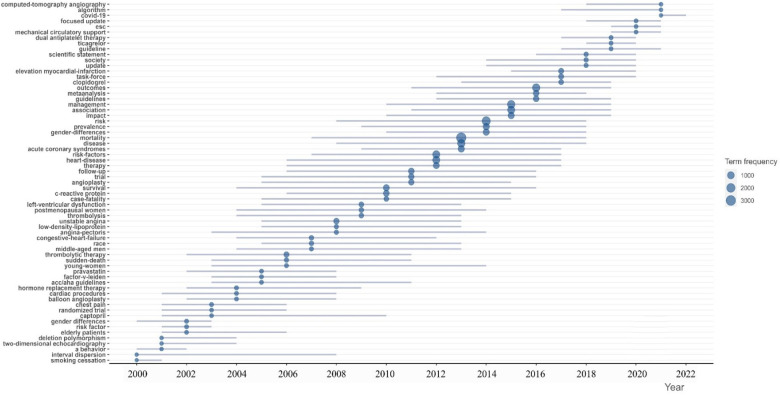
Trend topic analysis of researches about AMI in women.

The trend topic analysis of the keywords (Figure 10) showed that from 2000 to 2010, the research in this period mainly focused on risk factors, age, mortality, and therapy, while the main keywords were shown as smoking cessation, young women, balloon angioplasty, captopril, hormone replacement therapy, and sudden-death. Since 2011 scholars have begun to update the guidelines, and the main keywords changed to ticagrelor, dual antiplatelet therapy, society, outcomes, guidelines, focused update, covid-19, etc. Besides algorithm and appropriate use criteria have appeared frequently in recent years, which might represent the current research hotspots nowadays.

## Discussion

### General information

In the age of information explosion, bibliometric analysis can help researchers manage knowledge and visualize knowledge structures quickly and visually. The annual number and trend of literature would reﬂect the development speed and research progress of this study. While the number of articles published had increased in a steady growth phase from 414 in 2000 to the peak of 899 in 2021, which suggested that it had been one of the hot study directions in the cardiovascular field.

According to the distribution of countries/regions and institutions, the USA was shown as the country with the highest number of publications (5142, 34.60%), followed by England (1103, 7.42%), which would account for 42.02% of the total publications. Scholars from four countries were shown to keep close cooperations: USA, China, Canada, and Japan. Among the top ten research institutions, eight were from the United States, one from Canada, and one from Sweden. From the perspective of research institutions, most of the cooperations were limited to internal connections and the number of transnational cooperations was small. Therefore, institutions from various countries were suggested to carry out close cooperations and communications in order to promote the development of AMI in women.

Most of the research on AMI in women was published in *American Journal of Cardiology* (*n* = 522, 3.51%). Regarding co-cited academic journals, the majority of studies were published in high-impact Q1 journals, providing robust support for the ongoing study of AMI in women. Researches on AMI in women began to convert primary research results into clinical research, public health, and preventive medicine.

From the perspectives of author contributions and co-cited authors, Harlan M Krumholz (*n* = 110,0.74%) from Yale University is the author with the highest number of publications, followed by John A Spertus (*n* = 90, 0.61%), Eric D Peterson (*n* = 67, 0.45%), Roxana Mehran (*n* = 57, 0.38%), and Myung Ho Jeong (*n* = 47, 0.32%). It should be noted that Vaccarino V (0.09) exerts the signiﬁcant greatest publication impact and is the second most cited author. IN 2005, Vaccarino V and his colleagues found rates of reperfusion therapy, coronary angiography, and in-hospital death after myocardial infarction, but not the use of aspirin and beta-blockers, vary according to race and sex, with no evidence that the differences have narrowed in recent years (35). Recently, Vaccarino V and Zakaria A compared the association of mental stress–induced or conventional stress–induced ischemia with adverse cardiovascular events in patients with CAD and found that in patients with stable CAD, mental stress-induced ischemia was significantly associated with an increased risk of cardiovascular death or nonfatal myocardial infarction compared with no mental stress-induced ischemia (36). These findings may provide insights into the mechanisms of myocardial ischemia.

Among the top 10 co-cited references related to AMI in women, the top three co-cited references (37–39) focused on the effect of potentially modifiable risk factors, Sex-based differences in early mortality, and guidelines for the management. The most co-cited reference was published in European Heart Journal by Marco Roffi’s group (39). The authors showed guidelines for the management of acute coronary syndromes in patients presenting without persistent ST-segment elevation.

From the results of the above analysis, we found that the main authors, major research institutions and journals are from developed countries such as Europe and the United States, where mortality rates have reached a turning point as attention to female CAD has increased in these countries. In contrast, the Global Burden of Disease study reports that in most other parts of the world, this mortality rate remains stagnant with little or no change (40).

### The hotspots and frontiers

Keywords summarize research topics and core content. Based on keyword co-occurrence analysis, it is possible to understand the distribution and development of different research hotspots in a certain ﬁeld. In addition to AMI and women, the keywords that frequently appear in Table 5 are mortality (3592), risk factors (3163), outcomes (1463), disease (1383), association (1216), and prognosis (837). According to the trend topic analysis, algorithm and appropriate use criteria have appeared frequently in recent years. Then, we determine the research hotspots and development frontiers in the field of AMI in women. The main contents are as follows.

### Risk factors and disease

Although the traditional risk factors for cardiovascular disease are the same in women and men, the prevalence and impact of these risk factors differed between sex (41, 42). Due to the protective effect of estrogen and differences in risk factors, atherosclerosis was found to develop later in women than in men and was more common in patients of advanced age (43). Due to their advanced age, women with CAD usually had more comorbidities than men, such as diabetes, hypertension, chronic renal insufficiency, congestive heart failure, atrial fibrillation, and peripheral arterial disease. Low weight, malnutrition, anemia, fatigue, and debilitating states were more common in women with CAD due to menstrual cycle, co-morbidities, and advanced age (44, 45). Moreover, hypertension, diabetes, physical activity, and alcohol consumption had a greater impact on myocardial infarction in women under 60 years of age compared to men (38).

Data from the Virgo study suggested that young and middle-aged women hospitalized for type 1 and type 2 myocardial infarction were more likely to have lower socioeconomic status, higher psychosocial burden, such as depression and poorer physical/mental health, and overall lower quality of life compared with men (46). Thus, depression, trauma, and perceived stress had been identified as strong predictors of cardiovascular risk in young and middle-aged women (47, 48). In addition to the above all, low socioeconomic status was shown as an identified variable negatively associated with global coronary heart disease risk, with a higher additional risk in women compared with men (49). Emerging evidence suggested great sex differences in cardiovascular risk factors and comorbidities, including inflammatory processes, autoimmune diseases, cardiometabolic disorders, and major depression (44). Men and women have different genetic backgrounds, and energy and nutritional requirements throughout their lifespan. While differences in gastrointestinal transit times would lead to sex differences in gut flora (50). Indeed, systemic trimethylamine N-oxide levels—a metabolite produced by the gut microbiome—was shown to predict 30-day and 6-month event free survival in women and men with suspected ACS(44). Future studies might show the potential effects of novel diagnostic biomarkers and therapeutic targets based gut microbes in the evolution of intervention strategy against cardiovascular diseases especially in women.

Cardiovascular disease risk assessment remains challenging, especially in women. Although many predictive models exist, validity is often limited by small sample sizes and the specific characteristics of the populations from which they are derived. This results in lower validity of these tools for use in specific populations, including young women and minority populations. The use of sex-specific and age-specific cardiovascular disease risk thresholds and the incorporation of new measures of subclinical disease into risk assessment may improve guidance for preventive measures (51, 52).

Despite mandates by the National Institute of Health and the European funding body for inclusion of women in trials, women continue to be grossly underrepresented in Ischemic Heart Disease(IHD) trials (only 34% in 2006). Analysis of the 2007 women’s CVD prevention guidelines revealed that women comprised only 25% of the participants of the 156 IHD trials (53). Recognizing these disparities, the 2010 report published by the Institute of Medicine called for continued efforts to strengthen the inclusion of women in clinical trials (53). A thorough implementation of National Institute of Health mandates that ensure transparent and publicly available publication of sex-specific research data will be key.

### Outcome and prognosis

Female patients with AMI were shown to be at higher risk of serious complications, which might be associated with older patient age, more comorbid diseases, the tendency to delay diagnosis and treatment, the low rate of receiving reperfusion therapy, and the anatomical characteristics of the heart (54). Women with AMI were shown to have a higher Killip classification on admission, and are more likely to have acute complications such as cardiogenic shock, myocardial rupture, septal perforation, pericardial tamponade, and heart failure, and have a higher rate of bleeding as well as requiring transfusion therapy (53, 55). As a result, such vulnerable patients had longer hospital stays, a higher incidence of sudden death, and higher in-hospital, 30-day, and 1-year mortality rates than men (56). Prognostic factors for adverse outcomes after myocardial infarction in women were still elusive. And many risk prediction models based on the combination of parameters derived from medical history and clinical severity indicators, such as the GRACE (57)and TIMI (58) scores, are commonly used in ACS patients. However, these prediction models were most developed from a patient population that was at least two-thirds male, so they didn't perform well enough in female patients. For example, the hazard ratio for sinus rhythm deficits was 7.6 in women and 3.2 in men (59). Women with ST-segment elevation myocardial infarction (STEMI) without chest pain had a higher risk of in-hospital death in all age groups (60). The absence of chest pain appeared to be a stronger marker of mortality risk in women compared to men, especially in younger women (25). Compared to non-ST elevation myocardial infarction(NSTEMI), STEMI was also a more reliable short-term prognostic indicator for women than for men, with higher mortality within the first 24 h of hospitalization (18, 61). Diabetes is another strong prognostic factor, and its long-term risk of death was confirmed to be approximately doubled, which was greater for women than for men again (62, 63). Although age is a valid prognostic indicator for all patients after myocardial infarction (MI), the relationship between age and post-MI mortality was less pronounced in women than in men. The reason might be that women who experienced early myocardial infarction (<60 years old) had higher short-term mortality than men in the same age group, and this difference would decrease with increasing age (17, 37, 64). This disparity could be also seen after hospital discharge up to 2 years (65), but was less evident in the long-term follow-up more than 5 years (66). Women hospitalized for AMI had a high prevalence of cardiovascular risk factors, including hypertension, hypercholesterolemia, current smoking, diabetes, and obesity, all of which were confirmed prognostic indicators. In terms of psychological factors, women with MI, especially young women with early-onset MI, had a disproportionate burden of psychosocial risk factors despite similar or more favorable indicators of AMI severity compared with men of similar or older age (67, 68). Emerging evidence links psychosocial factors with poor outcomes in patients with ischemic heart disease, particularly depression, which is now a recognized prognostic factor of AMI (69). The prevalence of depression in post-MI patients is approximately 20%, several times higher than in the general population, and approximately twice as high in women with MI as in men (69). Depression was shown to be particularly common in younger women with MI (67, 68, 70).

### Criteria

In recent years, the paradigm of coronary atherosclerotic plaque rupture as the sole cause of STEMI or NSTEMI had been broken down, and evidence from intracoronary imaging showed that some cases of acute coronary syndromes were caused by plaque erosion rather than rupture (71, 72). Two conditions of particular interest in female patients were Takotsubo Syndrome (TTS) and spontaneous coronary artery dissection (SCAD). TTS would account for 1%–3% of all patients with suspected STEMI, more than 90% of all which occurred in women, especially menopausal women over 50 years of age (73). The main manifestations of TTS are shown as acute chest pain (75.9%), dyspnea (46.9%), and syncope (7.7%), most of which have negative emotional stress triggers (74). Left ventriculography is characterized by transient left ventricular dysfunction with or without apical involvement and localized ventricular wall motion abnormalities beyond the single coronary artery supply, usually without coronary angiographic evidence of obstructive coronary disease or acute plaque rupture, thrombosis, or vascular entrapment, but can coexist with obstructive coronary disease. TTS is often associated with new ECG abnormalities (ST-segment elevation and/or T-wave inversion) or mild elevation of cardiac troponin, which is less likely to be associated with serious complications, but some cardiovascular events such as myocardial rupture and sudden death can occur (75). The clinical presentation of TTS is highly similar to that of ACS, and it is difficult to distinguish them only by symptoms, which often need to be clarified by coronary angiography and left ventriculography and should be paid great attentions to female patients with acute chest pain. SCAD, a spontaneous separation of the coronary artery wall, forming an intramural hematoma and occluding the coronary lumen, mainly occurs in women, which predominates in younger women and is the most common cause of pregnancy-related myocardial infarction (76). It is vital that physician should take SCAD into consideration when facing women with suspected AMI.

The diagnosis of AMI depends on the clinical presentation, ECG findings, and biochemical evidence of myocardial injury. However, sex-based differences in the evaluation of AMI are also prevalent. Chest pain remains the most common clinical symptom in women with AMI, with more than 80% presenting with retrosternal pain, pressure, tightness, or discomfort (77). However, compared with men, women have more variable and less intense chest pain and more accompanying symptoms, such as nausea, vomiting, shortness of breath, weakness, excessive sweating, palpitations, and neck, shoulder, arm, jaw, or back pain (78). The VIRGO study showed that 61.9% of women under 55 years of age with myocardial infarction reported 3 or more concomitant symptoms compared to 54.8% of men (77). The proportion of women with myocardial infarction without chest pain symptoms was also significantly higher than that of men (42.0% vs. 30.7%), especially in younger women (<45 years) (25). There are significant sex differences in the serum thresholds for troponin, a specific marker of myocardial injury. It was found that cTn thresholds were significantly higher in men than in women (1.2–2.4 times), and the widespread clinical use of tests more applicable to men has resulted in compromised timeliness and specificity in the diagnosis of female myocardial infarction patients. In addition to cTn, ultrasensitive C-reactive protein and B-type natriuretic peptide, which are more likely to be elevated in women with heart attacks, are less often included in clinical risk stratification (79). Therefore, the use of sex-specific biomarker thresholds in clinical laboratory reports, as well as sex-differentiated risk stratification, can optimize individualized management of AMI, help physicians identify “female-pattern” AMI at early stage, and reduce the number of false negatives in female patients with high-risk chest pain (79).

### Algorithm

In the field of cardiovascular medicine, Artiﬁcial intelligence (AI) -based systems had been used in risk prediction, cardiovascular imaging, outcome prediction after operations, and new drug design. AMI is still one of the leading causes of death and demands huge health care expenditures worldwide. Risk scores such as the SYNTAX score (80) and the GRACE (81) are currently used as tools to predict major adverse cardiovascular events (MACE) in patients with previous or current AMI, but with not satisfactory clinical accuracy. Besides for patients with AMI, the cost of treatment usually depended on the extent of disease, pre-existing comorbidities, and the type and extent of revascularization procedures. Artificial intelligence (e.g., machine learning) could partially address and provide possible solutions to this unmet need (82). Predictive models with a wide range of health factors including comorbidities such as uncontrolled diabetes mellitus, or high blood pressure, dyslipidemia, socioeconomic factors, and angiographic factors would help the physicians to optimize the size of stents and the volume of contrast agent (83). Table 6 summarizes the application of AI in interventional cardiology. By using machine learning techniques, it is possible to precisely identify high-risk patients with high morbidity and mortality at early stage and to allocate and use of limited medical resources optimally.

**Table 6:** Summary of the studies representing the application of algorithm in ACS.

Rank	Author	Application of artiﬁcial intelligence
1	D'Ascenzo F	ML-based prediction of adverse events following an ACS (84).
2	McDaniel M	Algorithm in percutaneous coronary intervention capability (85).
3	Lu J	ML risk prediction model for ACS and death from use of non-steroidal anti-inflammatory drugs in administrative data (86).
4	Pieszko K	ML in predicting long-term mortality in patients with ACS (87).
5	Conde D	Algorithm for ACS using high-sensitivity troponin T assay vs fourth-generation troponin T assay (88).
6	Takeda M	ML in the diagnosis of ACS (89).
7	Kang JE	Development and clinical application of an evidence-based pharmaceutical care service algorithm in ACS (90).
8	Lan NSR	Implementing simple algorithms to improve glucose and lipid management in people with diabetes and ACS (91).
9	Banerjee A	ML for subtype definition and risk prediction in heart failure, ACS and atrial fibrillation (92).
10	Myers PD	ML Improves Risk Stratification After ACS (93).
11	Hernesniemi JA	Extensive phenotype data and ML in prediction of mortality in ACS (94).
12	Weichwald S	Improving 1-year mortality prediction in ACS patients using machine learning (95).
13	Emakhu J	ACS prediction in emergency care: A machine learning approach (96).
14	Berikol GB	Diagnosis of ACS with a Support Vector Machine (97).
15	Duan H	Utilizing dynamic treatment information for MACE prediction of ACS (98).
16	Liu Y	Beatquency domain and ML improve prediction of cardiovascular death after ACS (99).
17	Sherazi SWA	Artiﬁcial intelligence in the early prediction and diagnosis of major adverse cardiac events in patients with ACS (100).

ML, machine learning.

### Limitations

This study still contains some shortcomings. First off, other databases are neglected and only the WOSCC database is used for this study's data, which may leave out certain pertinent studies. Secondly, the quality of data collected in the literature varies, which may affect the credibility of the knowledge graph. Furthermore, the number of citations may depend on those authors working together rather than reflecting the quality of the original research. Some publications without citations do not necessarily have no scientific value. Also, some papers by these authors cannot be taken as higher scientific value than others not mentioned. Limited by the bibliometric methods, the citations were mainly used to grade the value of the articles, the conclusion should be interpreted with great cautions, and some other tool such as h-Index. might provide with more valuable information. Finally, since the enrolled papers included article and review, some of them are not specific to research in women and AMI, which might induce some bias into the final conclusions, but did not change the trend direction.

## Conclusions

AMI in women has important research value and application prospects. Visual analysis using CiteSpace and VOSviewer software has shown substantial trends in the study of AMI in women. The increasing number of articles published in international core journals indicated a significant impact. The leading countries were the United States and England; however, there is a need to strengthen collaboration and communication between experts and institutions from different countries. Nowadays researches on AMI in women focused on risk factors, disease, prognosis, mortality, criteria, and algorithm, which might still extend to the focus in the future.

## Data Availability

The original contributions presented in the study are included in the article, further inquiries can be directed to the corresponding author/s.
